# Age-Related Alterations of Hyaluronan and Collagen in Extracellular Matrix of the Muscle Spindles

**DOI:** 10.3390/jcm11010086

**Published:** 2021-12-24

**Authors:** Chenglei Fan, Carmelo Pirri, Caterina Fede, Diego Guidolin, Carlo Biz, Lucia Petrelli, Andrea Porzionato, Veronica Macchi, Raffaele De Caro, Carla Stecco

**Affiliations:** 1 Department of Neurosciences, Institute of Human Anatomy, University of Padua, 35121 Padua, Italy; chenglei.fan@studenti.unipd.it (C.F.); caterina.fede@unipd.it (C.F.); diego.guidolin@unipd.it (D.G.); lucia.petrelli@unipd.it (L.P.); andrea.porzionato@unipd.it (A.P.); veronica.macchi@unipd.it (V.M.); rdecaro@unipd.it (R.D.C.); 2 Orthopedics and Orthopedic Oncology, Department of Surgery, Oncology and Gastroenterology (DiSCOG), University of Padua, 35128 Padua, Italy; carlo.biz@unipd.it

**Keywords:** aging, extracellular matrix, muscle spindle, collagen, collagen type I, collagen type III, hyaluronan, intramuscular connective tissue

## Abstract

Background: Muscle spindles (MSs) play a crucial role in proprioception and locomotor coordination. Although the elasticity and viscosity of the extracellular matrix (ECM) within which MSs are embedded may play a key role in MS function, the impact of aging on ECM components is unclear. The aim of the current study was to investigate the age-related physiological changes of the ECM and to verify if these could be due to alterations of the environment directly surrounding MSs. Methods: Hematoxylin Eosin and picrosirius-red staining was carried out; collagen types I (COLI) and III (COLIII) were assessed, and biotinylated hyaluronan binding protein (HABP) immunohistochemical analysis was undertaken to evaluate alterations of the ECM in the intramuscular connective tissue (IMCT) of the hindlimbs of C57BL/6J male mice. Assessments were carried out on 6-week-old (Group A), 8-month-old (Group B), and 2-year-old (Group C) laboratory mice. Results: The capsule’s outer layer became progressively thicker with aging (it was 3.02 ± 0.26 μm in Group A, 3.64 ± 0.31 μm in Group B, and 5.81 ± 0.85 μm in Group C). The collagen in IMCT around and within the MSs was significantly higher in Group C, but there were no significant differences between Groups A and B. The MS capsules and continuous IMCT were primarily made up of COLI and COLIII. The average optical density (AOD) values of COLI in IMCT surrounding MS were significantly higher after aging (*p* < 0.05), but there were no significant differences in COLIII in the three groups (*p* > 0.05). HA was present in IMCT and filled the MSs capsule. The AOD of HABP of MS showed that there were lower HA levels in Group C with respect to Group A (*p* = 0.022); no significant differences were noted neither between Groups A and B nor between Groups B and C (*p* > 0.05). Conclusion: Age-related collagen accumulation and lower HA in the ECM in which the MSs were embedded may probably cause more stiffness in the ECM in vivo, which could help to partly explain the peripheral mechanisms underlying the age-related decline in functional changes related to MSs.

## 1. Introduction

Muscle spindles (MSs), which are detectors of muscle length and velocity, play an important role not only in proprioception but also in regulating muscle contraction [1,2]. Some time ago, a group of investigators [3] reported that the dynamic and static length sensitivities of MS primary endings in response to ramp stretch were decreased in aged rats. Age-related proprioceptive deficits have also been found to be associated with pathophysiological and morphological changes in MSs, which normally rapidly adapt to fibers in response to muscle length and speed changes. Possible morphological changes in MSs include: enlargement of the periaxial space [4], thicker spindle capsules [5,6,7,8], a lower number of intrafusal fibers [9], modifications in the myosin heavy chain content, lower sensitivity, smaller spindle diameters, changes in the shape of the MS primary endings (less helicity and vorticity), primary endings of aged MSs being less spiral or non-spiral in appearance [10], axonal swelling/expanded motor endplates due to denervation [11], and a significant increase in the number of Ia afferents with large swellings that fail to properly wrap around intrafusal muscle fibers [12]. Moreover, possible functional changes with MSs were also found. Aging is associated with alterations of MSs and their neural pathways. The muscle stiffness constant values were greater in old muscles, confirming the changes in elastic properties under passive conditions due to aging [13]. In addition, there are decreased conduction velocities and a less dynamic response of primary endings in old rats using electrophysiological experimentation [10]. Likewise, MSs containing thin muscle fibers may be intimately related to the degeneration and regeneration of extrafusal muscle fibers during aging, which may often fail to receive sensory innervation [14] and induce a decrease in the sensitivity, acuity, and integration of the proprioceptive signal [15].

New perspectives in this field recently became apparent when some investigators reported that even the intramuscular connective tissue (IMCT) of the extracellular matrix (ECM) in which MSs are embedded changes with aging [16]. The ECM refers to many proteins (collagen, elastic fibers) and ground substances (proteoglycans, multiadhesive glycoproteins, and glycosaminoglycans (GAGs)) that provide the milieu for the cells within the body [17]. Collagens are recognized as the main components of the ECM and form a family of 28 different types of collagens coded for by 44 different genes [18]. Proteoglycans can be categorized into four main families: small leucine-rich proteoglycans, hylectans, pericellular and basement membrane proteoglycans, and cell-surface proteoglycans [19]. Hyaluronan (HA) is the major extracellular GAG, which is found in many different tissues and has many roles, ranging from mechanical to chemical [19]. Cell behavior is profoundly affected by the environment in which they live, thus the aging of the ECM is central to understanding age-related changes [17]. Nowadays, ECM’s role has been highlighted in different diseases and tissues [19,20,21,22,23].

The different ECM elements provide the mechanical properties, plasticity, and malleability of the IMCT [24]. It has been reported, for example, that immobilization results in a marked increase in the collagen fibers in the IMCT (endomysium, perimysium, epimysium) and clearly disturb the normal IMCT structure of rat skeletal muscle. The changes in the IMCT in immobilized skeletal muscle seemed to contribute to alterations in the biomechanical properties of the tissue of skeletal muscle reducing its compliance [25]. This, in turn, increased the stretch reflex of the MS as the pull is transmitted more efficiently to the MSs in a less extensible muscle [26]. The MS is covered with a strong capsule of connective tissue that is continuous with the IMCT [27,28]. A gelatinous fluid rich in GAGs probably composed of primarily hyaluronan (HA) fills the inner and outer capsule spaces [29,30]. Although the elasticity and viscosity of the ECM within which MSs are embedded may play a key role in MS function, the impact of aging on ECM components is unclear.

In light of these considerations, we hypothesized that, beyond the normal neurodegeneration and morphological alterations of the MSs, age-related physiological changes could also be explained by alterations in the environment directly surrounding them. The study set out to investigate age-related changes in MSs and the ECM that could lead to altered functional outcomes.

## 2. Materials and Methods

Fifteen male C57Bl/6J mice (five 6-week-old mice = Group A, five 8-month-old mice = Group B, and five 2-year-old mice = Group C) were provided by the University of Padova’s Animal Center (Padova, Italy). The ages of the mice corresponded approximately to 11.5, 35, and 70 human years, respectively [31]. The mice were kept in cages in an environmentally controlled room with the temperature adjusted to 22 °C in which there were diurnal light-dark cycles and free access to water and food. The animals’ accommodation and care and all experimental procedures conformed to guidelines approved by the University of Padova’s Animal Care and Ethical Committee, in agreement with the guidelines of the Italian Department of Health.

Two percent isoflurane was used to anesthetize the mice. Following euthanasia, the left hindlimb (including the gastrocnemius; soleus; tibialis anterior; and posterior, fibularis longus, and brevis muscles) was collected for histological and immunohistochemistry studies. As hindlimb muscles in mice are extremely small and dissection can damage the IMCT in the skeletal muscles, the tibia and fibula bones were stored together to maintain the overall morphology and interrelationship of the hindlimb muscles and permit further specimen processing and experimental studies. The entire left hindlimb of each mouse was immediately post-fixed in 10% neutral buffered formalin (10% NBF, pH 7.4 at 4 °C for 48 h) to prepare for the decalcification procedure.

### 2.1. The Ethylenediaminetetraacetic Acid (EDTA) Decalcification Protocol for the Mice Hindlimbs

To preserve the antigenicity of the hindlimb sample, 10% EDTA solution at room temperature was used to decalcify the bone samples. All the samples post-fixed in 10% neutral-buffered formalin (NBF) were placed in phosphate buffered saline (PBS) (20 min × 3) and distilled water (20 min × 3). The fixed samples were then placed in 10% EDTA (at least 15 volumes), which was replaced weekly. The hindlimbs were decalcified in 10–14 days. The samples were then rinsed in distilled water (20 min × 2) for further paraffin embedding and histochemical and immunohistochemical staining.

After the samples were embedded in the paraffin wax, blocks were serially sectioned in a rostro-caudal direction and perpendicularly to the muscle fiber axis beginning under the lower edge point of the patella. Four sections at 100 μm intervals were transferred onto 2% gelatinized glass slides. A 100 μm section sampling interval was considered appropriate in light of the results of other studies [32,33], and a pilot study during which muscle tissue from two 6-week-old mice was longitudinally sectioned and the MS length was determined; it ranged between 180 and 400 μm. Sections of the muscle samples were subjected to routine histology staining (Hematoxylin Eosin, picrosirius-red) and the immunohistochemistry assessment of collagen type I (COLI), collagen type III (COLIII), and biotinylated hyaluronan binding protein (HABP) antibodies. The MS images at the equatorial region of the mice hindlimb muscle according their morphology were used to quantitatively analyze the components of the ECM in the MSs of the three mice groups.

### 2.2. Immunohistochemistry Staining: Analysis of Collagen Type I (COLI) and Collagen Type III (COLIII)

After formalin fixation, samples were dehydrated in graded ethanol, embedded in paraffin, and cut into 5 μm-thick sections. The sections were dewaxed in xylene 2 × 10 min and subsequently passed through 99–70–30% ethanol (10 min for each passage) and 2 × 5 min in water. Samples were treated with the blocking of endogenous peroxidases with 0.5% H2O2 in phosphate buffered solution (PBS; pH 7.4). The slides were then treated with 0.1% bovine serum albumin (BSA) for 1 h before being incubated with the primary antibody, the Anti-COLI, Anti-COLIII (Goat Anti-Collagen I: 1:400, SouthernBiotech, Birmingham, AL 35209, USA; Rabbit polyclonal to Collagen III: 1:400, ab7778 Sigma-Aldrich, Merk Life Science S.r.l., MI, Italy) in BSA at 4 °C overnight. After being washed three times with PBS, the sections were incubated with the secondary antibody (anti-rabbit IgG peroxidase-coniugated antibodies for COLIII and anti-Goat peroxidase-coniugated antibodies for COLI), 1:200, 1:300, respectively, for 1 h, after repeated washings; the reaction was developed with 3,3′-diaminobenzidine (Liquid DAB Substrate Chromogen System; Dako Corp, Carpinteria, CA, 93013-2921, USA). Negative controls were obtained by omitting the primary antibody. Finally, each slide was counterstained with hematoxylin, followed by dehydration in a graded ethanol series, and mounted for microscopic evaluation. The generation of the reaction product was stopped within the linear phase of the generation of the reaction product and to directly compare control (without primary antibody) with the three experimental tissues, leaving same the reaction time and solutions during the IHC [33].

### 2.3. Immunohistochemistry Staining: Analysis of Hyaluronic Acid Binding Protein (HABP)

Dewaxed 5 μm thick sections were treated with 0.5% H2O2 in PBS (15 min at room temperature) to block endogenous peroxidase and then washed in PBS. The specimens were incubated in 0.1% BSA solution for 1 h at room temperature, treated with biotinylated HABP (Millipore Sigma-Aldrich, Merk Life Science S.r.l., MI, Italy, 1:900 dilution), diluted in 0.1% BSA solution, and incubated overnight at 4 °C. After multiple washings with PBS, the samples were incubated with the secondary antibody, HRP-conjugated Streptavidin 1:250 for 30 min (Jackson ImmunoResearch, Cambridgeshire, UK) and washed in PBS buffer. The reaction was then developed with 3,3′-diaminobenzidine (Liquid DAB substrate Chromogen System kit Dako Corp, Carpinteria, CA, 93013-2921, USA) and it was terminated with distilled water. The nuclei were counterstained with ready-to-use hematoxylin (Dako Corp, Carpinteria, CA, 93013-2921, USA). Negative controls were checked with similarly treated sections, without the primary antibody, and the specificity of the immunostaining reaction was confirmed.

### 2.4. Image Analysis

The specimens were photographed using a Leica DMR microscope (Leica Microsystems, Wetzlar, Germany), and the images were analyzed with ImageJ software [34,35], which is freely available at http://rsb.info.nih.gov/ij/ (accessed on 18 August 2021).

**Figure 1:**
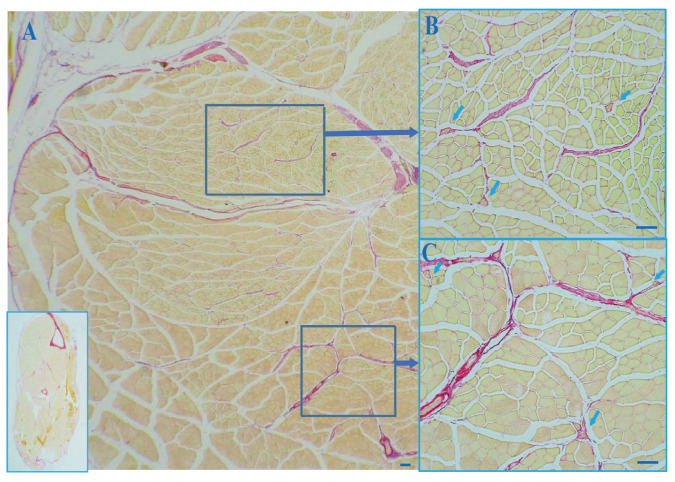
Picrosirius red staining of a cross section of a muscle of a mouse hindlimb. The inset in (**A**) on the bottom left corner is the whole crosse section of the mouse hindlimb. The MS capsule appears to be continuous with the perimysium, epimysium, and the endomysium. (**A**): Global view of the cross section. (**B**,**C**): MSs within the skeletal muscle. arrows: muscle spindles. Scale bar: 100 μm.

The thickness of the outer layer of the MS capsule and the collagen were measured using a final magnification of 40×. Fields containing MSs continuous with the perimysium and endomysium stained with picrosirius red were selected (Figure 1).

**Figure 2:**
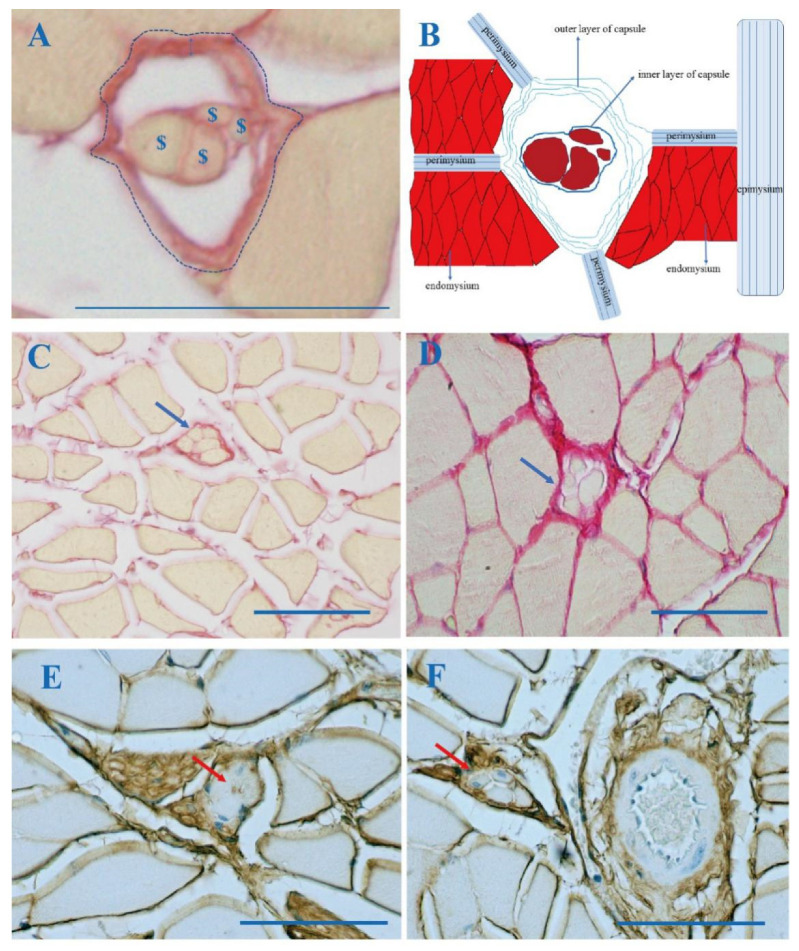
Picrosirius red (**A**,**C**,**D**) and biotinylated HABP immunohistochemistry staining (**E**,**F**) of a cross section of a muscle of a mouse hindlimb. (**A**): The MS in the skeletal muscle. $: intrafusal fiber; ↕: the thickness of the outer layer of the MS capsule; imaginary line: the area of the MS; (**B**): A drawing illustrating the MS’s outer capsule’s continuity with the endomysium, perimysium, and epimysium. (**C**): A MS in a muscle of the hindlimb of a 6-week-old mouse (Group A), (**D**): of a 2-year-old mouse (Group C). (**E**,**F**): HA is present in the MS capsule. The MS capsule was continuous with the nerve (**E**) and the blood vessel (**F**); arrows: muscle spindle; Scale bar: 50 μm.

A minimum of 30 images, including a MS from each mouse hindlimb, were obtained, and the data were averaged to calculate representative values for the thickness of the capsule’s outer layer, the area percentage of the total collagen in the cross sections of the muscle in which the MSs were embedded, and the area percentage of collagen in the MSs (Figure 2A,C,D). The results were expressed as the thickness of the outer layer of the capsule (μm) and the quantity of collagen in the MSs (%) per unit area.

The COLI, COLIII, and biotinylated HABP were measured using a final magnification of 40×. The average optical densities (AODs) of the COLI and COLIII of the area of the muscle cross sections in which MSs were embedded, of the area of the MS alone, and of the biotinylated HABP of the MS alone were measured. Average optical densities (AODs) = integrated optical density (IOD)/area. The semi-quantitative immunohistochemical images for analysis in the present study were taken with the same background, same exposition light for microscopy, and same background filter in Image J. In addition, the value changes with the intensity of the light source and is a part of the power ratio of the emission amplitude to the incident amplitude. In international application, the range of OD value is constant use, a scale of 0–2.71. Moreover, all the analyses are double-blinded. A minimum of 30 images was obtained, and the data regarding the AOD were averaged to calculate the representative values for the COLI, COLIIII, and the biotinylated HABP. Considering that differences in any parameter need to be limited to approximately the same area of the same muscle, a minimum of 30 MS images each are from tibialis anterior, peroneus longus, peroneus brevis (10 MS images); gastrocnemius, soleus (10 MS images) and flexor digitorum longus, flexor hallucis longus, and tibialis posterior (10 MS images).

### 2.5. Statistical Analysis

All data management and statistical analyses were performed using IBM SPSS version 25.0 software (SPSS Inc., Chicago, IL, USA). The Shapiro–Wilks and Levene’s tests were respectively performed to investigate the normality of the data distribution and the homogeneity of variance. The thickness of the capsule’s outer layer, the area percentage of the total collagen in the muscle cross sections in which MSs were embedded, the area percentage of the collagen in the MS, and the AOD of the COLI and of the COLIII in the entire muscle cross sections in which MSs were embedded and in the MS alone are reported as means ± standard deviations (M ± SD) since they were found to have a normal distribution. An analysis of variance (ANOVA) with Tukey post-hoc test (normally distributed data and equal variances assumed) was carried out to compare the AOD of the COLI and the COLIII of the area of the entire muscle cross sections where the MSs were embedded and the AOD of the area of the MS alone to investigate age-related effects on collagen and subtypes (COLI, COLIII). In addition, the ANOVA with the post-hoc test of Games–Howell (normally distributed data but equality of variances not assumed) were both used to compare the thickness of the outer layer of the capsule, the area percentage of the total collagen in the entire muscle cross section in which the MSs were embedded, and the area percentage of collagen in the MS alone. The AOD of the biotinylated HABP of the MS was classified as median, minimum, and maximum, since its distribution was not normal. The Kruskal–Wallis H with Bonferroni post-hoc test was used to compare the AOD of the biotinylated HABP of the MSs of the three groups. A p-value of less than 0.05 was considered the study’s limit for statistical significance.

## 3. Results

### 3.1. The Outer Capsule of MS Is Continuous with Intramuscular Connective Tissue (IMCT)

All the MSs were embedded in the IMCT, and the outer spindle capsules were found to be continuous with the endomysium, perimysium, and epimysium (Figure 1 and Figure 2B). In addition, the outer spindle capsule was continuous with the nerve (Figure 2E) and the blood vessel (Figure 2F). The integrity of and the continuity between the different muscles and between different MSs within the same and different muscles were maintained by the IMCT.

### 3.2. Collagen in the MS with Aging

**Figure A1:**
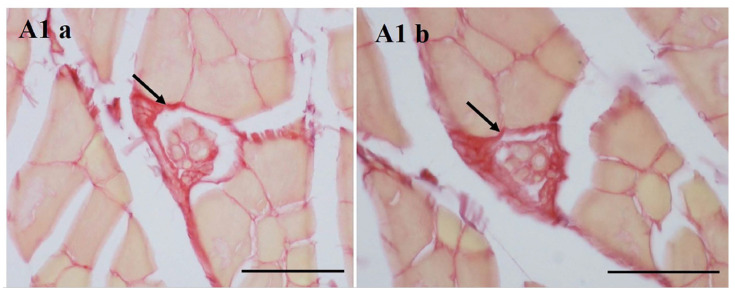
(a:b) MS embedded in the extracellular matrix elements with picrosirius red (**a**,**b**) of the hindlimb of an 8-month-old mouse (Group B). Scale bar: 50 μm.

MSs are surrounded by a strong capsule found in the IMCT between the extrafusal fibers. The MS capsule contains outer and inner layers. The thickness of the capsule’s outer layer was 3.02 ± 0.26 μm in the adolescent, 3.64 ± 0.31 μm in the middle-aged, and 5.81 ± 0.85 μm in the elderly mice. In addition, the area percentage of the total collagen in the entire cross section in which the MSs were embedded was 2.95 ± 0.46 in the adolescent, 4.42 ± 1.23 in middle-aged, and 9.29 ± 0.81 in the elderly mice. With regard to the MS alone, the area percentage of collagen was 22.97 ± 6.55 in the adolescent, 25.94 ± 2.36 in the middle-aged, and 40.80 ± 3.46 in the elderly mice. The thickness of the capsule’s outer layer, the area percentage of the total collagen in the entire muscle cross sections in which the MSs were embedded, and the area percentage of collagen in the MS alone were significantly higher in Group C (the elderly group) with respect to Group A (the adolescent one) (*p* < 0.01) and Group B (the middle-aged group) (*p* < 0.01); there were no significant differences, except for the the thickness of the capsule’s outer layer, in these parameters between Groups A and B (*p* > 0.05), (*p* = 0.022) (Table 1, Figure 2A,C,D and Figure A1).

### 3.3. COLI and COLIII in MS

**Figure 3:**
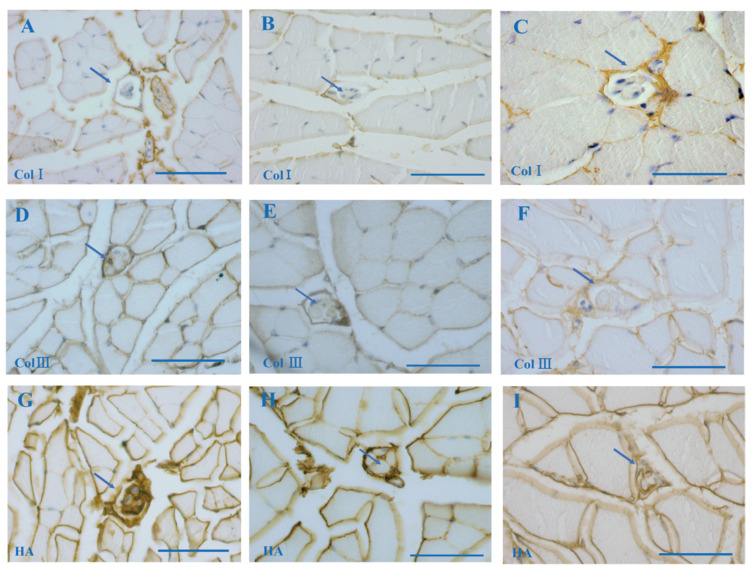
MS embedded in the extracellular matrix elements with immunohistochemistry staining of collagen type I (COLI) (**A**–**C**), collagen type III (COLIII) (**D**–**F**), and biotinylated HABP (**G**–**I**). The COLI and COLIII localized in the MS capsule. The HABP localized in the MS. (**A**,**D**,**G**): A MS in the hindlimb of a 6 week-old mouse (Group A); (**B**,**E**,**H**): a MS in the hindlimb of an 8-month-old mouse (Group B); (**C**,**F**,**I**): a MS of the hindlimb of a 2-year-old mouse (Group C). Scale bar: 50 μm.

Collagen fibers, as fundamental components of ECM, provide a supporting framework of muscle tissues. In this study, immunohistochemistry staining uncovered that the MS capsule and the IMCT consisted of COLI and COLIII (Figure 3A–F). The AOD of COLI in the whole cross section in which MSs were embedded was 0.20 ± 0.02 in the adolescent, 0.27 ± 0.02 in the middle-aged, and 0.30 ± 0.01 in the elderly mice. The AOD of COLI in the MS alone was 0.16 ± 0.01 in the adolescent, 0.22 ± 0.01 in the middle-age, and 0.28 ± 0.06 in the elderly mice. The AOD of COLI in the whole muscle cross section in which the MSs were embedded and the AOD of COLI in the MS alone were significantly increased with aging (Group A vs. Group B: *p* < 0.05; Group A vs. Group C: *p* < 0.001; Group B vs. Group C: *p* < 0.05). The AOD of COLIII in the whole muscle cross section was 0.25 ± 0.02 in the adolescent, 0.28 ± 0.01 in the middle-aged and 0.26 ± 0.02 in the elderly mice. The AOD of COLIII in the MS alone was 0.25 ± 0.07 in the adolescent, 0.27 ± 0.07 in the middle-aged, and 0.26 ± 0.05 in the elderly mice. There were no significant differences in the COLIII neither in the whole muscle cross section in which MSs were embedded nor in the MS alone between the three groups (*p* > 0.05) (Table 1).

### 3.4. Age-Related HA in MS According to Biotinylated HABP Immunohistochemical Staining

**Figure A2:**
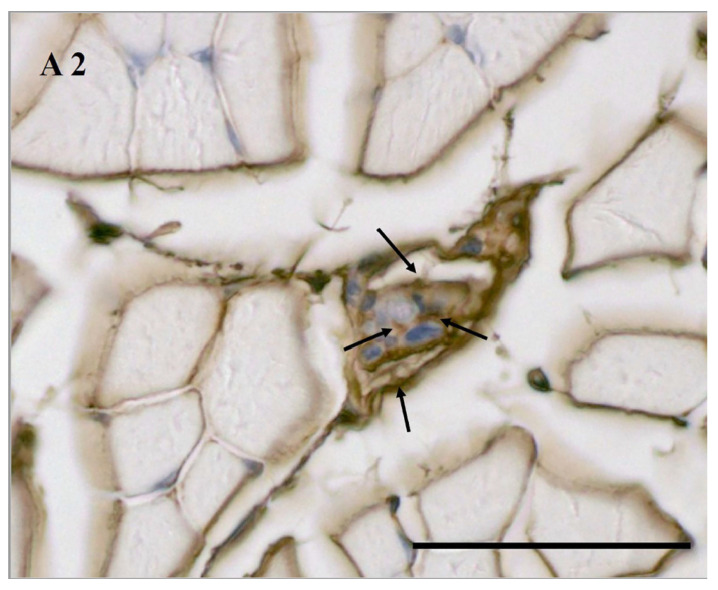
HA is present in the MS capsule and round intrafusal fibres. Scale bar: 50 μm.

The ground substance of the ECM is composed of a complex mixture GAGs, most often covalently linked to proteins, forming proteoglycans and glycoproteins, in which HA appears to be the one of the most important ones. In the present study, HA was present in the IMCT (epimysium, perimysium, epimysium) and filled the MS capsule according to the biotinylated HABP immunohistochemistry staining (Figure 3E,F and Figure A2). The AOD of the MS in the hindlimb muscle was significantly decreased in Group C (0.40, 0.33–0.47) (Figure 3I) with respect to Group A (0.50, 0.45–0.67) (Figure 3G) (*p =* 0.022); no significant differences neither between Groups A and B (0.43, 0.40–0.45) (Figure 3H) nor between Groups B and C (*p* > 0.05) were noted (Table 1).

## 4. Discussion

The study results outlined here have demonstrated that MSs are embedded in the IMCT and that the MS outer capsule is continuous with the perimysium, epimysium, endomysium, nerve, and blood vessel. Other studies have already demonstrated the continuity of the outer capsule with the ECM of extrafusal fibers in chicken [36] and with the perineural epithelium [37]. IMCT acts as the scaffold for muscle bundles and fiber integrity and carries the blood vessels and nerves to the muscle. IMCT continuity permits communication between different muscles, various MSs within the same muscle, and various MSs in different muscles directly and/or indirectly. This communication may play an important role in movement coordination. In addition, the intrafusal fibers of MSs possess intracapsular terminations or extend beyond the limit of the MS capsule terminating in the IMCT of adjacent extrafusal fibers [38,39,40]. MS distribution seems to be similar to the three-dimensional arrangement and organization of the IMCT from the fascia point of view (IMCT belongs to the fascial system). If MSs are viewed not only as isolated mechanoreceptors but also as sensory organs embedded and enclosed in the IMCT, this would explain how they can sense the tension of the IMCT as far as the fascia are concerned.

Study results also demonstrated that the capsule’s outer layer, the area percentage of the total collagen in the whole cross section in which MSs were embedded and the collagen in the MSs were significantly thicker in the elderly mice and that there were no significant differences in these parameters, with the exception of the thickness of the capsule’s outer layer, between the adolescent and middle-aged mice. This is in line with other studies about the thicker capsule’s outer layer by Swash and Fox (1972) [9]. An analysis of our results also showed that the MS capsule and the IMCT consisted of COLI and COLIII. While the AOD of the COLI in the whole cross section where the MSs were embedded and the AOD of COLI in the MS alone were significantly increased in the aging mice, there were no significant differences in COLIII in the three groups neither in the entire cross section in which MSs were embedded nor in the MS alone. These results have confirmed the findings of another study demonstrating that older adults have thicker MS capsules [11]. Moreover, these findings are consistent with those of some studies that highlighted the role of the collagens in various diseases and tissues [21,22,23]. Parkes et al. [21] reported the prominent role of collagen and HA in the ECM of ovaries, suggesting their crucial activity for ovarian homeostasis, possibly through signaling events or tissue micromechanism. Numerous reports described the association between the single-nucleotide polymorphism rs12722 and rs13936 in the COL5A1 gene and injuries, such as Achilles tendon pathology, anterior cruciate ligament, and tennis elbow [41,42,43,44,45]. Daleswski et al. [22] studied the COL5A1 rs12722 and rs13946 polymorphisms as potential genetic factors regulating the ADDwoR-mediated soft tissue pathway in association with temporomandibular joint anterior disc displacement [22]. Pirri et al. [46] reported that:” excessive aggregation of ECM elements is present in fibrosis (e.g., in myopathies), including in fasciae, and occurs during aging [16], and diabetes, characterized by increased endomysium as well as perimysium” [47]. Diet-induced insulin resistance (IR) leads to an increase in the expression of collagens I, III, and IV [48].

However, the MS are sensitive to both the phasic and tonic stretches. When the muscle lengthens and the MS is stretched, the threshold corresponds to a tension of 3 g in humans and leads to a trigger action in the MS afferent [49]. The accumulation of collagen, especially of the COLI type, due to aging (found in elderly mice) may reduce the deformation and increase the resistance of the IMCT in the skeletal muscle, resulting in a higher threshold.

HA was found to be present in the IMCT and filled the MS capsule. Study results showed that the HA in the MS was significantly decreased in the elderly mice with respect to their adolescent counterparts. There were, however, no significant differences between the elderly and middle-aged mice nor between the middle-aged and adolescent mice. HA is not only an excellent lubricant and shock absorber [24], but also on the background discharge and the discharge in response to stretch [50]. The age-related decrease in HA could affect the mechanical properties of MSs, the background discharge, and the discharge in response to stretch, which may in turn affect age-related changes in MSs’ sensitivity to the stretch and tension of skeletal muscles. As has been demonstrated by other studies, HA has many roles in the different human tissues, ranging from mechanical to chemical [19,21,48,51].

MSs also play an important role in reactive postural control, and they are also involved in producing contractile force during reflectory changes. The MSs may be unable to adapt to stretching and velocity due to the age-related alterations of the ECM in the skeletal muscle resulting in failed motor unit recruitment. Fewer motor unit activities may lead to a lower contractile strength of the muscle [52,53]. MSs also seem to participate in regulating sensitivity during the dynamic and/or static phases of stretching; this would mean that, as they become less sensitive with aging, there is a decline in postural stability and balance. During flexion of the trunk, for example, the MSs of the erector spinae muscles are lengthened. This stimulates the recruitment of motor units, resulting in the contraction of the muscles and helping the trunk return to the starting position. The same mechanism is found in the neck and other body segments [54,55]. If MSs become less sensitive to stretching due to aging, trauma, poor posture, post-surgery, or overuse, this could exacerbate changes in sensory and motor functions, leading to greater postural instability and a higher risk of falls, and the inhibition of normal MS stretching could also result in abnormal feedback to the central nervous system.

There are several limitations in this study. Firstly, aging-related changes in the musculoskeletal system included a decline not only involved in the alterations of HA and collagen in the ECM of the MSs but also a reduction in the number of MSs per muscle, a loss of intrafusal fibers, and changes in the efficacy of the fusimotor innervation. In addition, the age-related decline of the velocity of action potential propagation by sensory neurons, sarcopenia, changes in the structure of the sensory nerve terminal, and the loss of neurons in the motor cortex and cerebellum have already been demonstrated. All of these changes could potentially contribute to the decline in motor coordination, frequent falls, and unstable gait observed in elderly persons. Moreover, the inner and outer capsule contain many other ECM proteins as described in the introduction, including other proteins: laminins, nidogens, collagen type IV, fibronectin, agrin, and other proteoglycans. These molecules could also affect the biomechanical properties of the spindle capsule. Moreover, the treatment of histological sections involves tissue dehydration and thus, shrinkage. As reported by other studies [56,57], the tissue shrinkage takes place during the preparation of connective tissue specimens for histological examination. At the same time, there is likely no difference in shrinkage between young and old, as reported by Kerns et al. [58], as the decrease of shrinkage was relatively constant across age groups. Further studies are necessary to better illustrate these factors with aging, to better understand the effects of aging on locomotor ability decline.

## 5. Conclusions

As we found that a change in the thickness of the outer capsule, the decreased staining intensity with HA and increased staining intensity with of Col I in ECM may probably cause the MSs themselves and the surrounding microenvironment to experience more stiffness with aging in vivo. Other studies regaridng functional changes in MS have already demonstrated age-related muscle stiffness and elastic properties [13] and decreased conduction velocities and dynamic response of primary endings with aging [10]. Moreover, MSs contained thin muscle fibers that may be intimately related to the degeneration and regeneration of extrafusal muscle fibers during aging, which may often fail to receive sensory innervation [14], and that may induce a decrease in the sensitivity, acuity, and integration of the proprioceptive signal [15], which may also reduce the sensitivity of the MSs and their ability to activate motor neurons stimulating muscle contraction and to contribute to postural maintenance and positional sense, as well as to maintaining muscle tone. It has been seen, in fact, that, when the MSs cannot be activated, the regulation of muscle tone is compromised [59]. These alterations in ECM where MSs are embedded could help to explain partly the peripheral mechanisms underlying the age-related decline in functional changes related to MS.

## Figures and Tables

**Table 1:** Collagen constituting the ECM of the MSs in the mouse skeletal muscle.

Characteristic	Group A	Group B	Group C	A vs. B *p*-Value	A vs. C *p*-Value	B vs. C *p*-Value
^$^ Thickness of the outer capsule layer	3.02 ± 0.26	3.64 ± 0.31	5.81 ± 0.85	0.022 *	0.003 **	0.007 **
^$^ Total collagen 40× (% area)	2.95 ± 0.46	4.42 ± 1.23	9.29 ± 0.81	0.116	<0.001 ***	<0.001 ***
^$^ Collagen of the MS alone 40× (% area)	22.97 ± 6.55	25.94 ± 2.36	40.80 ± 3.46	0.633	0.004 **	<0.001 ***
^#^ AOD of COLI in the whole muscle cross section	0.20 ± 0.02	0.27 ± 0.02	0.30 ± 0.01	<0.001 ***	<0.001 ***	0.032 *
^#^ AOD of COLIII in the whole muscle cross section	0.25 ± 0.02	0.28 ± 0.01	0.26 ± 0.02	0.255	0.747	0.629
^#^ AOD of COLI in the MS alone	0.16 ± 0.01	0.22 ± 0.01	0.28 ± 0.06	0.038 *	<0.001 ***	0.047 *
^#^ AOD of COLIII in the MS alone	0.25 ± 0.07	0.27 ± 0.07	0.26 ± 0.05	0.849	0.968	0.950
^&^ AOD of HABP in the MS alone	0.50 (0.45–0.67)	0.43 (0.40–0.45)	0.40 (0.33–0.47)	0.85	0.022 *	1.00

ANOVA with Tukey post-hoc test ^#^/Games–Howell post-hoc test ^$^; Kruskal–Wallis H with Bonferroni post-hoc test ^&^, MS: muscle spindle, AOD: average optical density, Group A: equivalent to human adolescence (6-week-old mice), Group B: equivalent to human middle age (8-month-old mice), Group C: equivalent to old age in humans (2-year-old mice) group. The values are presented as numbers or means ± SD (normally distributed data and equal variances assumed); the values are classified as median, minimum, and maximum, since their distribution was not normal. * <0.05, ** ≤0.01, *** ≤0.001.

## Data Availability

The data presented in this study are available on request from the corresponding author.

## Abbreviations

Muscle spindles: MSs; extracellular matrix: ECM; collagen type I: COLI; collagen type III: COL III; hyaluronan binding protein: HABP; intramuscular connective tissue: IMCT; glycosaminoglycans: GAGs; average optical density: AOD; hyaluronan: HA.
